# Transformation of Tri-Titanium(IV)-Substituted α-Keggin Polyoxometalate (POM) into Tetra-Titanium(IV)-Substituted POMs : Reaction Products of Titanium(IV) Sulfate with the Dimeric Keggin POM Precursor under Acidic Conditions

**DOI:** 10.3390/ma3010503

**Published:** 2010-01-15

**Authors:** Yuki Mouri, Yoshitaka Sakai, Yoshitaka Kobayashi, Shoko Yoshida, Kenji Nomiya

**Affiliations:** Department of Materials Science, Faculty of Science, Kanagawa University, Hiratsuka, Kanagawa 259-1293, Japan

**Keywords:** Keggin polyoxometalate (POM), titanium(IV)-substitution, Ti-O-Ti bonding dimer, host-guest chemistry, three host-four guest coordination, sulfate coordination

## Abstract

Reaction products of titanium(IV) sulfate in HCl-acidic aqueous solution with the dimeric species linked through three intermolecular Ti-O-Ti bonds of the two tri-titanium(IV)-substituted α-Keggin polyoxometalate (POM) subunits are described. Two novel titanium(IV)-containing α-Keggin POMs were obtained under different conditions. One product was a dimeric species through two intermolecular Ti-O-Ti bonds of the two tetra-titanium(IV)-substituted α-Keggin POM subunits, *i.e.*, [[{Ti(H_2_O)_3_}_2_(μ-O)](α-PW_9_Ti_2_O_38_)]_2_^6-^ (**1**). The other product was a monomeric α-Keggin species containing the tetra-titanium(IV) oxide cluster and two coordinated sulfate ions, *i.e.*, [{Ti_4_(μ-O)_3_(SO_4_)_2_(H_2_O)_8_}(α-PW_9_O_34_)]^3-^ (**2**). Molecular structures of **1** and **2** were also discussed based on host (lacunary site)-guest (titanium atom) chemistry.

## 1. Introduction

Polyoxometalates (POMs) are molecular metal-oxide clusters, which have attracted considerable attention in the fields of catalysis, medicine, surface science and materials science, since POMs are often considered as molecular analogues of metal oxides in term of structural analogy [1,2,3,4,5,6,7,8]. Site-selective substitution of the W^VI^ atoms in POMs with Ti^IV^ atoms is particularly interesting, because of formation of the multicenter active sites with corner- or edge-sharing TiO_6_ octahedra and, also, generation of oligomeric species through Ti-O-Ti bonds [9,10,11,12,13,14,15,16,17,18]. A number of catalytic reactions of titanium(IV)-containing POMs have also been reported so far [19,20,21].

The ionic radius of Ti^IV^ (0.75 Å) is close to that of W^VI^ (0.74 Å), suggesting that Ti^IV^ should fit nicely into the POM framework. However, there is a significant consequence in terms of oligomeric Ti-O-Ti anhydride formation resulting from substitution by several Ti^IV^ atoms. For instance, tri-Ti^IV^-1,2,3- and di-Ti^IV^-1,2-substituted Keggin POMs heretofore reported have been isolated as dimeric, Ti-O-Ti bridged anhydride forms, *e.g.*, [(β-1,2,3-SiW_9_Ti_3_O_37_)_2_O_3_]^14-^ [9], [(α-1,2,3-GeW_9_Ti_3_O_37_)_2_O_3_]^14-^ [10], [(β-1,2,3-GeW_9_Ti_3_O_37_)_2_O_3_]^14-^ [11], [(α-1,2,3-PW_9_Ti_3_O_37_)_2_O_3_]^12-^ [12], and [(α-1,2-PW_10_Ti_2_O_38_)_2_O_2_]^10-^ [13]. Di-Ti^IV^-substituted γ-Keggin silicotungstate and germanotungstate, *i.e.*, [{γ-SiTi_2_W_10_O_36_(OH)_2_}_2_O_2_]^8-^ [14] and [{γ-GeTi_2_W_10_O_36_(OH)_2_}_2_O_2_]^8-^ [15], have been also prepared as a dimeric species, while di-Ti^IV^-1,5-substituted β-Keggin POM has been isolated as a tetrameric species, [{β-Ti_2_SiW_10_O_39_}_4_]^24-^ [16]. In addition, the cyclic tri-Ti^IV^-substituted Keggin trimers such as [(α-Ti_3_PW_9_O_38_)_3_(PO_4_)]^18-^ [17], [(α-Ti_3_SiW_9_O_37_OH)_3_(TiO_3_(OH_2_)_3_)]^17-^ [17], and {K[(Ge(OH)O_3_) (GeW_9_Ti_3_O_38_H_2_)_3_]}^14-^ [18] have been recently reported.

From the viewpoint of host-guest chemistry of Ti-substitution in POM, in contrast to most Ti^IV^-substituted Keggin POMs consisting of a combination of a mono-lacunary site (one host) and an octahedral Ti group (one guest), an unusual host-guest relationship has been found in some recent POMs, [[{Ti(ox)(H_2_O)}_4_(μ-O)_3_](α-PW_10_O_37_)]^7-^ (H_2_ox = oxalic acid) **3** [22] and [{Ti(ox)(H_2_O)}_2_(μ-O)](α-PW_11_O_39_)]^5-^ **4** [23]. The two host-four guest POM **3** [22] in the solid state is composed of four octahedral Ti groups (four guests), *i.e.*, the two intramolecular Ti-O-Ti bonds linked with the μ-O atom, incorporated to the two adjacent, octahedral vacant sites (two hosts) within the di-lacunary Keggin POM. This POM has been prepared by a 4:1 molar-ratio reaction in HCl-acidic solution (pH 0.08) of the anionic titanium(IV) complex [TiO(ox)_2_]^2-^ with the dimeric form of the two di-titanium(IV)-substituted, α-Keggin POM units, K_10_[(α-1,2-PW_10_Ti_2_O_39_)_2_]·18H_2_O [13]. The one host-two guest POM **4** [23] has been also obtained by the reactions under strongly acidic conditions of [TiO(ox)_2_]^2-^ with mono- or tri-lacunary Keggin POMs, [α-PW_11_O_39_]^7-^ or [A-PW_9_O_34_]^9-^, and also with the dimeric species of the two mono-titanium(IV)-substituted Keggin POM units, [(PW_11_TiO_39_)_2_OH]^7-^ as precursors. The last reaction was based on the finding of **4** in solution, *i.e.*, its dissociation equilibrium to [(PW_11_TiO_39_)_2_OH]^7-^, [TiO(ox)_2_]^2-^ and H^+^. In preparations of **3** and **4**, it should be also noted that the dimeric species of the two mono-and/or di-titanium(IV)-substituted Keggin POM units, but not the lacunary Keggin POMs, can be used as POM precursors.

In this work, to further extend such reactions, we have investigated the reaction products of Ti(SO_4_)_2_·4H_2_O with [(α-1,2,3-PW_9_Ti_3_O_37_)_2_O_3_]^12-^ [12] as the dimeric precursor linked through three intermolecular Ti-O-Ti bonds of the two tri-titanium(IV)-substituted α-Keggin POM units under aqueous HCl-acidic conditions. Two novel titanium(IV)-containing α-Keggin POMs were synthesized under different conditions; one was K_5_H[[{Ti(H_2_O)_3_}_2_(μ-O)](α-PW_9_Ti_2_O_38_)]_2_·9H_2_O (**K-1**) identified as the dimeric species through the two intermolecular Ti-O-Ti bonds of the two tetra-titanium(IV)-substituted Keggin POM units, and the other was K_3_[{Ti_4_(μ-O)_3_(SO_4_)_2_(H_2_O)_8_}(α-PW_9_O_34_)]·6H_2_O (**K-2**), characterized as the monomeric species containing tetra-titanium(IV) oxide cluster and two coordinated sulfate ions (*note:* the corresponding polyoxoanion moieties are abbreviated simply as **1** and **2**, respectively). These compounds were unequivocally characterized with complete elemental analysis, TG/DTA, FT-IR, X-ray crystallography, solid-state ^31^P-CPMAS NMR and solution (^31^P-, ^183^W-) NMR spectroscopy. Herein, we report full details of the synthesis, characterization and host-guest chemistry of the novel POMs, **K-1** and **K-2**.

## 2. Experimental Section

### 2.1. Materials

The following reactants were used as received: KCl, 1 M aqueous HCl solution (quantitative analysis grade) (all from Wako); Ti(SO_4_)_2_·4H_2_O (Junsei); D_2_O (Isotec). The POM precursor, K_10_H_2_[(α-1,2,3-PW_9_Ti_3_O_37_)_2_O_3_]·xH_2_O (x = 17 [12], 15), was prepared according to the literature and identified by FT-IR, TG/DTA and ^31^P-NMR.

### 2.2. Instrumentation/Analytical Procedures

Complete elemental analysis was carried out by Mikroanalytisches Labor Pascher (Remagen, Germany). The sample was dried at room temperature under 10^-3^–10^-4^ Torr overnight before analysis. Infrared spectra was recorded on a Jasco 4100 FT-IR spectrometer in KBr disks at room temperature. Thermogravimetric (TG) and differential thermal analyses (DTA) were acquired using a Rigaku Thermo Plus 2 series TG/DTA TG 8120 instrument. TG/DTA measurement was run under air with a temperature ramp of 4 °C/min between 20 and 500 °C.

^31^P{^1^H}-NMR (161.70 MHz) spectra in D_2_O solution were recorded in 5-mm outer diameter tubes on a JEOL JNM-EX 400 FT-NMR spectrometer with a JEOL EX-400 NMR data processing system. ^31^P-NMR spectra were measured in aqueous solution with reference to an external standard of 25% H_3_PO_4_ in H_2_O in a sealed capillary. The ^31^P-NMR signals were shifted to +0.544 ppm by using 85% H_3_PO_4_ as a reference instead of 25% H_3_PO_4_. ^183^W-NMR (16.50 MHz) spectra were recorded in 10-mm outer diameter tubes on a JEOL JNM-EX 400 FT-NMR spectrometer equipped with a JEOL NM-40T10L low-frequency tunable probe and a JEOL EX-400 NMR data-processing system. ^183^W-NMR spectra measured in D_2_O were referenced to an external standard of saturated Na_2_WO_4_-D_2_O solution. The ^183^W-NMR signals were shifted to –0.787 ppm by using a 2 M Na_2_WO_4_ solution as a reference instead of saturated Na_2_WO_4_ solution.

Solid-state ^31^P-CPMAS NMR (121.00 MHz) spectra were recorded in 6-mm outer diameter tubes on a JEOL JNM-ECP 300 FT-NMR spectrometer with a JEOL ECP-300 NMR data-processing system and were referenced to an external standard, (NH_4_)_2_HPO_4_. Chemical shift is reported as negative for resonance upfield of (NH_4_)_2_HPO_4_ (δ 1.60).

### 2.3. Synthesis

#### 2.3.1. K_5_H[[{Ti(H_2_O)_3_}_2_(μ-O)](α-PW_9_Ti_2_O_38_)]_2_·9H_2_O (**K-1**)

Ti(SO_4_)_2_·4H_2_O (0.15 g, 0.48 mmol) was dissolved in a vigorously stirred aqueous 1 M HCl solution (10 mL). To the colorless, clear solution was added K_10_H_2_[(α-1,2,3-PW_9_Ti_3_O_37_)_2_O_3_]·15H_2_O (0.50 g, 0.09 mmol). This solution was stirred for 10 min in a water bath at 60 °C. To the clear solution solid KCl (0.75 g, 10.0 mmol) was added. After cooling to room temperature, the colorless clear solution was allowed to stand overnight in a refrigerator at 4 °C. The colorless plate crystals formed were collected on a membrane filter (JG 0.2 μm), washed with ice-cooled water (5 mL) and dried *in vacuo* for 2 h. The white powder obtained in 25.6% yield (0.13 g scale) was soluble in water and insoluble in most organic solvents containing EtOH and Et_2_O. Anal. {Found: H, 0.47; K, 3.39; O, 26.70; P, 1.12; Ti, 7.07; W, 61.40; total 100.15%. Calc. for H_25_K_5_O_90_P_2_Ti_8_W_18_ or K_5_H[[{Ti(H_2_O)_3_}_2_(μ-O)](α-PW_9_Ti_2_O_38_)]_2_ : H, 0.47; K, 3.61; O, 26.59; P, 1.14; Ti, 7.07; W, 61.11%}. A weight loss of 2.40% (weakly solvated or adsorbed water) was observed during the course of drying at room temperature at 10^-3^–10^-4^ Torr overnight before analysis, suggesting the presence of 7–8 water molecules. TG/DTA under atmospheric conditions: a weight loss of 6.69% was observed at below 200.0 °C; calc. 6.79% for total of 21 water molecules, *i.e.*, 12 coordinated water molecules plus *x* = 9 hydrated water molecules in K_5_H[[{Ti(H_2_O)_3_}_2_(μ-O)](α-PW_9_Ti_2_O_38_)]_2_·*x*H_2_O. IR (KBr) (poly-oxometalate region): 1,097 s, 1,045 s, 960 vs, 895 s, 795 vs, 692 vs, 582 w, 516 w, 497 w cm^-1^; Solid-state ^31^P-NMR: δ −9.54; ^31^P-NMR (22.9 °C, D_2_O): δ −9.70; ^31^P-NMR (22.1 °C, 0.1 M HCl *aq*.): δ −9.61.

#### 2.3.2. K_3_[{Ti_4_(μ-O)_3_(SO_4_)_2_(H_2_O)_8_}(α-PW_9_O_34_)]·6H_2_O (**K-2**)

K_10_H_2_[(α-1,2,3-PW_9_Ti_3_O_37_)_2_O_3_]·15H_2_O (2.0 g, 0.36 mmol) was added to a clear colorless solution of Ti(SO_4_)_2_·4H_2_O (0.60 g, 1.92 mmol) dissolved in 1 M aqueous HCl solution (40 mL). The colorless clear solution was stirred for 30 min in a water bath at 80 °C. To the solution was added solid KCl (0.20 g, 2.68 mmol). After cooling to room temperature, the clear solution was evaporated at 40 °C to a volume of *ca*. 5 mL with a rotary evaporator. The resulting white suspension was stored in a refrigerator at 4 °C overnight. The white precipitate formed was filtered off through a membrane filter (JG 0.2 μm). The clear filtrate was slowly evaporated at room temperature. After three days, clear colorless plate crystals formed, which were used for X-ray diffraction measurement. The remaining crystals were collected on a membrane filter (JG 0.2 μm) and dried *in vacuo* for 2 h. The colorless plate crystals obtained in 40.3% yield (0.88 g scale) were soluble in water and insoluble in most organic solvents. Stability in water is low; it decomposes within a few days at room temperature to produce several unknown materials. Anal. {Found: H, 0.55; K, 4.48; O, 29.5; P, 1.02; S, 2.38; Ti, 6.52; W, 54.80; total 99.25%. Calc. for H_16_K_3_O_53_P_1_S_2_Ti_4_W_9_ or K_3_[{Ti_4_(μ-O)_3_(SO_4_)_2_(H_2_O)_8_}(α-PW_9_O_34_)]: H,0.55 ; K, 4.01; O, 29.01, P, 1.06; S, 2.19; Ti, 6.55; W, 56.61%}. A weight loss of 3.83% (weakly solvated or adsorbed water) was observed during the course of drying at room temperature at 10^-3^–10^-4^ Torr overnight before analysis, suggesting the presence of 6 water molecules. TG/DTA under atmospheric conditions: a weight loss of 7.92% was observed at below 201.0 °C; calc. 7.78% for total of 14 water molecules, *i.e.*, 8 coordinated water molecules plus *x* = 6 hydrated water molecules in K_3_[{Ti_4_(μ-O)_3_(SO_4_)_2_(H_2_O)_8_}(α-PW_9_O_34_)]·*x*H_2_O. IR (KBr) (polyoxometalate region) 1,232 m 1,200 w 1,128 m 1,092 s, 1,028 m, 962 s, 926 s, 793 vs, 607 m, 519 m, 476 m cm^-1^; Solid-state ^31^P-NMR: δ −14.43; ^31^P-NMR (22.4 °C, D_2_O): δ −14.96; ^31^P-NMR (23.4°C, 0.5 M H_2_SO_4_ *aq*.): δ −15.07. ^31^P-NMR (22.5 °C, 1 M HNO_3_ *aq*.): δ −14.86; ^183^W-NMR (22.0 °C, 0.5 M H_2_SO_4_ *aq*.): δ −120.8 (3W × 1), −179.1 (6W × 1).

### 2.4. X-Ray Crystallography

A colorless plate crystal of **K-1** (0.29 × 0.08 × 0.03 mm) and a colorless plate crystal of **K-2** (0.23 × 0.18 × 0.14 mm) were surrounded by liquid paraffin (Paratone-N) to prevent their degradation. Data collection was done by Bruker SMART APEX CCD diffractometer at 90 K in the range of 1.15 ° < *θ* < 28.30 ° (**K-1**) and 1.15 ° < *θ* < 28.34 ° (**K-2**). The intensity data were automatically collected for Lorentz and polarization effects during integration. The structure was solved by direct methods (program SHELXS-97) [24] followed by subsequent difference Fourier calculation and refined by full-matrix, least-square procedure on *F*^2^ (program SHELXL-97) [25]. Absorption correction was performed with SADABS (empirical absorption correction) [26]. The composition and formula of the POM containing many counterions and many hydrated water molecules have been determined with complete elemental analysis and TG/DTA analysis. Refinements of the positions and temperature factors of many solvent molecules and countercations in the POM are limited because of their disorder. Consequently, the residual electron densities in the final difference maps for **1** and **2** were rather large. We can reveal only the molecular structure of the POM, but not the crystal structure. These features are too common in the POM crystallography [9,10,11,12,13,14,15,16,17,18].

#### 2.4.1. Crystal data for **K-1**

H_43_K_5_O_99_P_2_Ti_8_W_18_, *M* = 5,577.28, triclinic, space group *P-1*, *a* = 11.8044 (10), *b* = 13.7073 (12), *c* = 18.5727 (16) Å, *α* = 72.0090 (10), *β* = 84.4570 (10), *γ* = 65.245 (2) °, *V* = 2,593.7 (4) Å^3^, Z = 1, *D*_c_ = 3.571 Mg m^-3^, *μ*(Mo-Kα) = 20.781 mm^-1^. *R*_1_ = 0.0606, w*R*_2_ = 0.1465 (for all data). *R*_int_ = 0.0333, *R*_1_ = 0.0553, w*R*_2_ = 0.1429, GOF = 1.110 (25,624 total reflections, 12,729 unique reflections where *I* > 2*σ*(*I*)). The maximum and minimum residual density (9.764 and −5.468 eÅ^-3^) holes were located at 2.79 Å from O(4W) and 0.69 Å from W(3), respectively. The polyoxoanion **1** consisting of 18 tungsten atoms, eight titanium atoms, two phosphorus atoms, and 90 oxygen atoms, including the oxygen atoms due to 12 coordinated water molecules and two μ-O atoms, and five potassium cations treated with a disorder model, per formula unit, were identified, but the location of nine hydrated water molecules per formula unit were not determined as a result of disorder. CSD number 421237.

#### 2.4.2. Crystal data for **K-2**

H_28_K_3_O_59_PS_2_Ti_4_W_9_, *M* = 3,030.86, monoclinic, space group *P*2_1_/*c*, *a* = 17.8107 (11), *b* = 13.4314 (9), *c* = 23.1442(15) Å, *β* = 93.5690(10) °, *V* = 5525.9(6) Å^3^, Z = 4, *D*_c_ = 3.643 Mg m^-3^, *μ*(Mo-Kα) = 19.646 mm^-1^. *R*_1_ = 0.0404, w*R*_2_ = 0.1012 (for all data). *R*_int_ = 0.0348, *R*_1_ = 0.0354, w*R*_2_ = 0.0981, GOF = 1.043 (51,341 total reflections, 13,749 unique reflections where *I* > 2*σ*(*I*)). The maximum and minimum residual density (6.443 and −2.312 eÅ^-3^) holes were located at 2.74 Å from O(8W) and 0.78 Å from W(3), respectively. The polyoxoanion **2** consisting of nine tungsten atoms, four titanium atoms, one phosphorus atom, two sulfur atoms, and 53 oxygen atoms, including the oxygen atoms due to the eight coordinated water molecules, two coordinated sulfate ions, per formula unit, were identified. The location of three potassium cations treated with a disorder model and six hydrated water molecules, per formula unit, were also identified. CSD number 421238.

## 3. Results and Discussion

### 3.1. Synthesis and Compositional Characterization

The water-soluble potassium salts **K-1** and **K-2** were obtained in 25.6% (0.13 g scale) and 40.3% (0.88 g scale) yields, respectively, under the different conditions using approximately 1:5-molar ratio solution of K_10_H_2_[(α-1,2,3-PW_9_Ti_3_O_37_)_2_O_3_]·15H_2_O and Ti(SO_4_)_2_·4H_2_O. Compound **K-1** was derived by a 10-min reaction at 60 °C, while **K-2** was obtained by a 30-min reaction at 80 °C. The crystalline samples were unequivocally characterized by complete elemental analysis including O analysis, TG/DTA, FT-IR, solid-state and solution ^31^P-NMR spectroscopy and X-ray crystallography. The formation of polyoxoanions **1** and **2** can be represented in Equations (1) and (2):

[(α-1,2,3-PW_9_Ti_3_O_37_)_2_O_3_]^12-^ + 2Ti(SO_4_)_2_ + 13H_2_O → [[{Ti(H_2_O)_3_}_2_(μ-O)](α-PW_9_Ti_2_O_38_)]_2_^6-^ (**1**) + 4SO_4_^2-^ + 2H^+^ (1)

[(α-1,2,3-PW_9_Ti_3_O_37_)_2_O_3_]^12-^ + 2Ti(SO_4_)_2_ + 13H_2_O + 6H^+^ → 2[{Ti_4_(μ-O)_3_(SO_4_)_2_(H_2_O)_8_}(α-PW_9_O_34_)]^3-^ (**2**) (2)

For complete elemental analysis the two samples, **K-1** and **K-2**, were dried at room temperature under a vacuum of 10^-3^–10^-4^ Torr overnight. All elements (H, K, O, P, Ti and W for **K-1** and H, K, O, P, S, Ti and W for **K-2**) were observed for a total analysis of 100.15% for **K-1** and 99.25% for **K-2**. The data we found were in good accord with the calculated values for the formula with five potassium cations, 12 coordinated water molecules, two μ-O groups and without any hydrated water molecules for **K-1**, and for the formula with three potassium cations, eight coordinated water molecules, three μ-O groups, two coordinated sulfate ions and without any hydrated water molecules for **K-2** (see Experimental section). The weight losses observed during drying before analysis were 2.40% for **K-1** and 3.83% for **K-2**, which corresponded to *ca.* 7–8 and six hydrated water molecules, respectively. Thus, the elemental analyses indicated a presence of a total of 19–20 water molecules for **K-1** and of a total of 14 water molecules for **K-2** under atmospheric conditions.

On the other hand, in the TG/DTA measurements carried out under atmospheric conditions, the weight loss of 6.69% was observed at below 200.0 °C for **K-1** and that of 7.92% was observed at below 201.0 °C for **K-2**. The former value corresponded to a total of *ca*. 21 water molecules (calc. 6.79%) for **K-1**, which were assigned to 12 coordinated water molecules plus nine hydrated water molecules, whereas the latter corresponded to a total of *ca*. 14 water molecules (calc. 7.78%) for **K-2**, which were assigned to eight coordinated water molecules plus six hydrated water molecules. Thus, the total water molecules observed by TG/DTA measurements under atmospheric conditions are approximately agreed with the total water molecules found by elemental analysis. The formulas presented herein are based on the results of TG/DTA measurements. 

**Figure 1:**
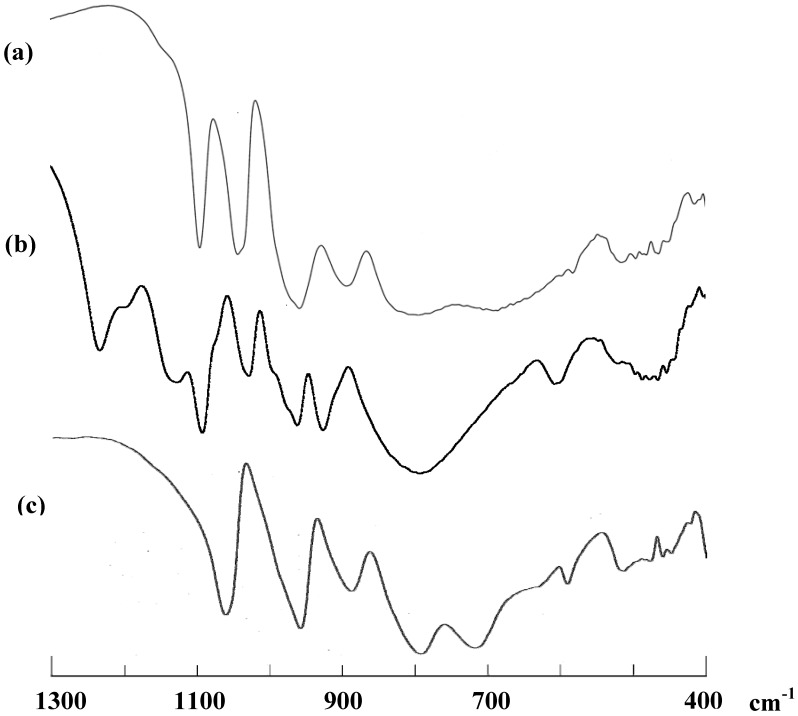
The FT-IR spectra in the region (1,300–400 cm^-1^), measured as KBr disks, of (a) K_5_H[[{Ti(H_2_O)_3_}_2_(μ-O)](α-PW_9_Ti_2_O_38_)]_2_·9H_2_O (**K-1**), (b) K_3_[{Ti_4_(μ-O)_3_(SO_4_)_2_(H_2_O)_8_}(α-PW_9_O_34_)]·6H_2_O (**K-2**), in which the coordinated sulfates are seen as bands at 1,232, 1,200 and 1,128 cm^-1^, and (c) K_10_H_2_[(α-1,2,3-PW_9_Ti_3_O_37_)_2_O_3_]·15H_2_O as a precursor.

**Figure 2:**
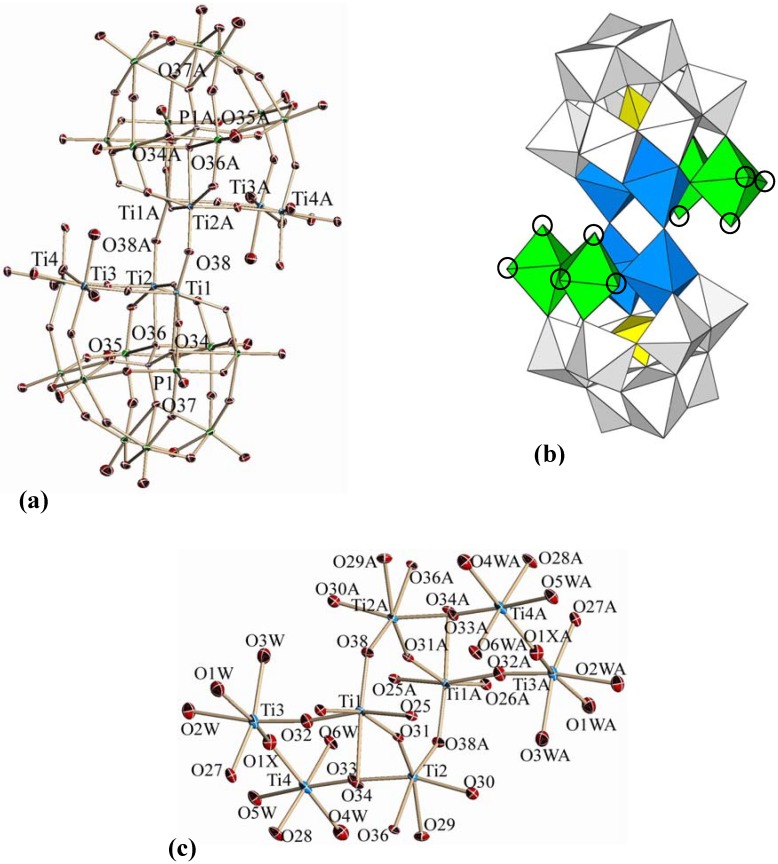
(a) Molecular structure of the dimeric polyoxoanion [[{Ti(H_2_O)_3_}_2_(μ-O)](α-PW_9_Ti_2_O_38_)]_2_^6-^ (**1**) in **K-1**, (b) its polyhedral representation, and (c) the partial structure around the Ti_8_ center. In (b), the four octahedral groups (green) of the eight octahedral TiO_6_ units are in the class of one host-two guest type coordination and the remaining four octahedral groups (blue) are in the class of one host-one guest type coordination. The two intermolecular Ti-O-Ti bonds are formed between the octahedra (blue octahedra) of the one host-one guest coordination. All 18 WO_6_ octahedra are shown in gray and the two central PO_4_ groups are shown in yellow. Total 12 coordinated water molecules are exhibited as open circles in the terminal positions in the four TiO_6_ (green) octahedra.

The solid FT-IR spectra, measured as KBr disks, of **K-1** (Figure 1a), **K-2** (Figure 1b) and the precursor K_10_H_2_[(α-1,2,3-PW_9_Ti_3_O_37_)_2_O_3_]·15H_2_O (Figure 1c), showed the characteristic vibrational bands of Keggin-type “PW_12_O_40_^n-^” polyoxotungstate framework (Figure 1) [27]. In the FT-IR spectra of **K-1** and the precursor, the bands based on the Ti-O-Ti vibration between the two Keggin units are observed at 692 and 718 cm^-1^, respectively, suggesting that they are dimeric species, while in the spectrum of **K-2** no Ti-O-Ti vibrational band was observed, suggesting that it is monomeric species. In the spectrum of **K-2**, bands due to the coordinated sulfate ions are observed at 1,232, 1,200 and 1,128 cm^-1^.

### 3.2. Molecular Structures of ***1*** and ***2***

The molecular structure of polyoxoanion **1** in K-**1**, its polyhedral representation, and the partial structure around the Ti_8_ center are shown in Figure 2a, Figure 2b and Figure 2c, respectively. Selected bond lengths (Å) and angles (°) around the Ti_8_ center in **1** are given in Table 1, while other bond lengths (Å) and angles (°) in **1** (Table S1) and the bond valence sum (BVS) calculations of the W, Ti, O and P atoms (Table S3) are deposited in the Supplementary material.

The composition and formula of **K-1** containing five potassium counterions, 12 coordinated water molecules and nine hydrated water molecules were determined by complete elemental analysis and TG/DTA analysis. In X-ray crystallography, polyoxoanion **1**, five potassium cations treated with a disorder model and 12 hydrated water molecules based on BVS calculations, per formula unit, were identified in the crystal structure (see Experimental section), but the location of nine hydrated water molecules per formula unit were not determined as a result of disorder.

The molecular structure of **1** is composed of two “PW_9_Ti_4_O_45_” Keggin POM halves linked via two Ti-O-Ti bonds between them. Each half contains the Ti_4_ center consisting of two Ti atoms (blue octahedra) of one host (mono-lacunary site)-one guest (one Ti atom) coordination and two Ti atoms (green octahedra) of one host-two guest coordination. The Ti_4_ center is composed of four corner-sharing Ti octahedra. The two Ti_4_ centers are linked through the two Ti-O-Ti bonds, each bond being formed between the two Ti atoms (blue octahedra) of one host-one guest coordination. In each Keggin POM half unit, six coordinated water molecules shown by BVS calculations occupy the six terminal positions of the two Ti atoms (green octahedra) of one host-two guest coordination, which are shown as open circles in Figure 2b. The whole symmetry of the molecule is represented by point group *C*_2h_.

Bond angles around the Ti_8_ center of **1** can be compared with those around the Ti_6_ center of the precursor POM [(α-1,2,3-PW_9_Ti_3_O_37_)_2_O_3_]^12-^ [12]; (1) the two Ti-O-Ti bonds between the two Keggin units of **1** [136.0(5)°] vs the three Ti-O-Ti bonds between the two Keggin units of the precursor [131.0(7)–131.3(7)°] and (2) the four Ti-O-Ti bonds within the Keggin unit of **1** (between two blue octahedra [155.7(5)°], between two green octahedra [136.9(5)°], and between one blue and one green octahedra [165.9(6)–169.8(6)°]) vs the three Ti-O-Ti bonds within the Keggin unit of the precursor [141.7(7) –147.4(8)°].

Bond lengths around the Ti_8_ center of **1** can be also compared with those around the Ti_6_ center of the precursor. The Ti-O lengths in the two Ti-O-Ti bonds between the two Keggin units [1.806(9)−1.832(9), average 1.819 Å], and the Ti-O lengths in the four Ti-O-Ti bonds within the Keggin units [1.747(9)−1.915(9), average 1.839 Å] of **1**, which have been obtained at the present refinement, can be compared with those of the precursor, *i.e.*, the Ti-O lengths in the three Ti-O-Ti bonds (between the two Keggin units) [1.80(1)−1.86(1), average 1.83 Å] and the Ti-O lengths in the three Ti-O-Ti bond lengths (within the Keggin units) [1.79(1)−1.92(1), average 1.87 Å]. 

In both **1** and the precursor, the W-Ot (Ot: terminal oxygen), W-Oc (Oc: corner sharing oxygen), W-Oe (Oe: edge-sharing oxygen), and W-Oa (Oa: oxygen coordinated to P atom) lengths were almost the same and in the normal range [2].

**Figure 3:**
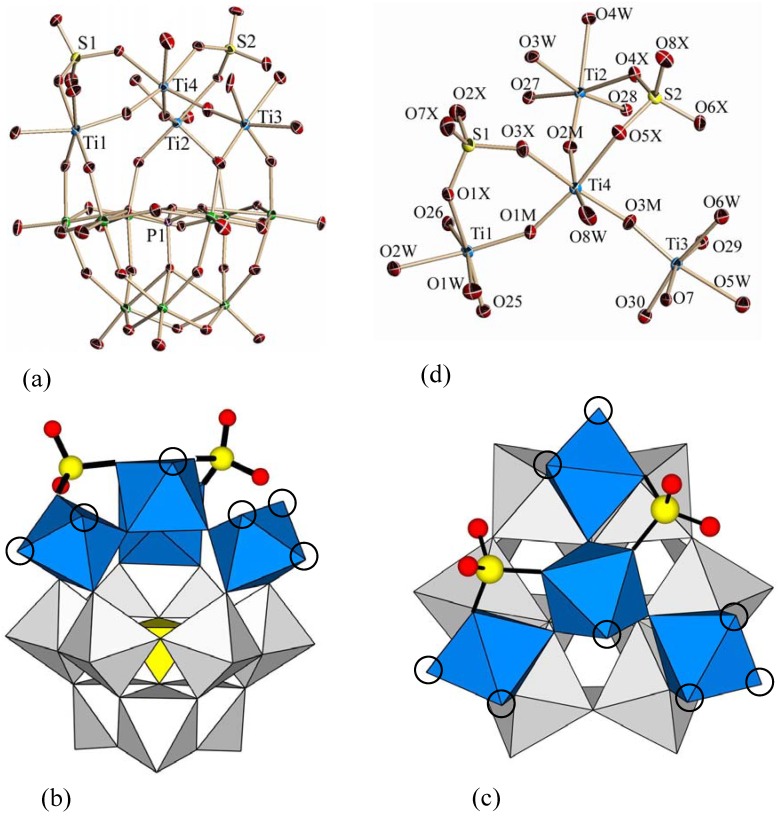
(a) Molecular structure of the monomeric polyoxoanion [{Ti_4_(μ-O)_3_(SO_4_)_2_(H_2_O)_8_}(α-PW_9_O_34_)]^3-^ (**2**) in **K-2**, polyhedral representations ((b) side view and (c) top view), and (d) the partial structure around the Ti_4_ center. In (b) and (c), the four octahedral TiO_6_ units are shown in blue, all nine WO_6_ octahedra are shown in gray, and the one central PO_4_ group is shown in yellow. The two sulfate ions coordinated to titanium(IV) octahedra are shown as yellow circles (S) and red circles (O), and the coordinated water molecules to titanium(IV) octahedra are exhibited by open circles.

The molecular structure of polyoxoanion **2** in **K-2**, its polyhedral representation (side view and top view), and the partial structure around the Ti_4_ center are shown in Figure 3a, (Figure 3b and Figure 3c) and Figure 3d, respectively. Selected bond lengths (Å) and angles (°) around the Ti_4_ centers in **2** are also given in Table 1, while other bond lengths (Å) and angles (°) in **2** (Table S2) and the bond valence sum (BVS) calculations of the W, Ti, S, O and P atoms (Table S4) are deposited in the Supplementary material.

The composition and formula of **K-2** containing three potassium counterions, eight coordinated water molecules and six hydrated water molecules were determined by complete elemental analysis and TG/DTA analysis. In X-ray crystallography, polyoxoanion **2**, three potassium cations treated with a disorder model, eight coordinated water molecules based on BVS calculations and six hydrated water molecules, per formula unit, were identified in the crystal structure (see Experimental section).

**Table 1:** Selected bond lengths (Å) and angles (**°**) around the titanium(IV) centers in **K-1** and **K-2**.

**lengths**	**K-1**		**K-2**
Ti(1)-O(25)	2.014(9)	Ti(1)-O(25)	1.871(6)
Ti(1)-O(26)	2.001(9)	Ti(1)-O(26)	1.813(6)
Ti(1)-O(31)	1.823(9)	Ti(1)-O(1M)	1.823(6)
Ti(1)-O(32)	1.912(9)	Ti(1)-O(1W)	2.154(6)
Ti(1)-O(34)	2.382(9)	Ti(1)-O(2W)	2.100(6)
Ti(1)-O(38)	1.806(9)	Ti(1)-O(1X)	2.069(6)
average	1.990	average	1.972
Ti(2)-O(29)	2.053(9)	Ti(2)-O(27)	1.898(5)
Ti(2)-O(30)	1.974(9)	Ti(2)-O(28)	1.888(6)
Ti(2)-O(31)	1.803(9)	Ti(2)-O(2M)	1.745(5)
Ti(2)-O(33)	1.915(9)	Ti(2)-O(3W)	2.097(6)
Ti(2)-O(36)	2.294(9)	Ti(2)-O(4W)	2.139(5)
Ti(2)-O(38)*^i^*	1.832(9)	Ti(2)-O(4X)	2.053(5)
average	1.9785	average	1.970
Ti(3)-O(27)	1.907(10)	Ti(3)-O(29)	1.873(6)
Ti(3)-O(32)	1.753(9)	Ti(3)-O(30)	1.860(6)
Ti(3)-O(1X)	1.897(10)	Ti(3)-O(3M)	1.749(6)
Ti(3)-O(1W)	2.080(11)	Ti(3)-O(5W)	2.135(6)
Ti(3)-O(2W)	2.179(10)	Ti(3)-O(6W)	2.124(6)
Ti(3)-O(3W)	2.101(11)	Ti(3)-O(7W)	2.166(7)
average	1.9862	average	1.9845
Ti(4)-O(28)	1.871(10)	Ti(4)-O(1M)	1.791(6)
Ti(4)-O(33)	1.747(9)	Ti(4)-O(2M)	1.862(6)
Ti(4)-O(1X)	1.864(10)	Ti(4)-O(3M)	1.860(6)
Ti(4)-O(4W)	2.119(11)	Ti(4)-O(8W)	2.088(6)
Ti(4)-O(5W)	2.162(9)	Ti(4)-O(3X)	2.082(6)
Ti(4)-O(6W)	2.097(9)	Ti(4)-O(5X)	2.102(5)
average	1.9767	average	1.9642
angles	**K-1**		**K-2**
Ti(1)-O(31)-Ti(2)	155.7(5)	Ti(1)-O(1M)-Ti(4)	153.0(4)
Ti(1)-O(32)-Ti(3)	169.8(6)	Ti(2)-O(2M)-Ti(4)	153.0(3)
Ti(2)-O(33)-Ti(4)	165.9(6)	Ti(3)-O(3M)-Ti(4)	164.8(4)
Ti(3)-O(1X)-Ti(4)	136.9(5)		
Ti(1)-O(38)-Ti(2)*^i^*	136.0(5)		

Symmetry operations: *i* = -x+1,-y+1,-z+1.

The molecular structure of **2** is the monomeric POM composed of the Ti_4_ cluster accompanied with the two coordinated sulfate ions constructed on the tri-lacunary Keggin unit. Regardless of the coordination of the sulfate ions, an arrangement of the Ti_4_ cluster can be considered as a new type of host-guest relation, *i.e.*, three host (tri-lacunary site)-four guest (four Ti octahedra) coordination. Although, in **1**, the Ti_4_ center contained in the Keggin POM half unit may be also considered as a kind of three host-four guest coordination, it is only a combination of two Ti^IV^ atoms based on one host-one guest coordination and two Ti^IV^ atoms based on one host-two guest coordination. The previously reported, two host-four guest coordination can be also reconsidered as a combination of one host-two guest coordination [22]. The two sulfate ions bridged between the terminal positions of two Ti octahedra (central Ti4 octahedron and peripheral Ti1 or Ti2 octahedron). In total, eight coordinating water molecules in **2** are assigned to two each on the two peripheral Ti1 and Ti2 octahedra, one on the central Ti4 octahedron and three on the peripheral Ti3 octahedron without the coordination of sulfate ion, which are shown as open circles in Figure 3b and Figure 3c. Thus, whole symmetry of this molecule is exhibited by point group *C*_1_, *i.e.*, POM **2** takes chiral configuration. In fact, an enantiomeric pair was found in the unit cell.

Two bonds (Ti2-O2M-Ti4 and Ti1-O1M-Ti4) of three Ti-O-Ti bonds containing the central Ti4 octahedron are almost equivalent and distinguished from the remaining one (Ti3-O3M-Ti4 bond), which contains a Ti3 octahedron without coordination to the sulfate ion. This fact is reflected on the angles [153.0(4), 153.0(3) and 164.8(4)°]. However, the Ti-O lengths in all Ti-O-Ti bonds were almost the same. As a matter of fact, the Ti-O lengths in the two almost equivalent Ti-O-Ti bonds (Ti2-O2M-Ti4 and Ti1-O1M-Ti4) were in the range of 1.745(5)−1.862(6) Å, while those of the remaining Ti3-O3M-Ti4 bond were in the range of 1.749(6)−1.860(6) Å. Thus, coordination of sulfate ion affects the bond angles of Ti-O-Ti, rather than the bond lengths.

The bond valence sum (BVS) calculations [28,29] (Table S3 and Table S4), based on the observed bond lengths, suggest that all atoms, except for the six doubly protonated oxygen atoms (O1W-O6W: 0.374–0.489) in **1** and the eight doubly protonated oxygen atoms (O1W-O8W: 0.387–0.478) in **2**, *i.e.*, both due to water molecules, maintain formal valences (W^6+^, Ti^4+^, P^5+^, S^6+^ and O^2-^). In **1** and **2**, no protonation was confirmed in any Ti-O-Ti bonds between the Keggin units and, also, within the Keggin units. In **2**, BVS values of the oxygen atoms bonded to sulfur atoms (O1X-O8X: 1.500–1.943) suggest formal valence O^2-^, *i.e.*, no protonation to sulfate ions.

### 3.3. Solid-State and Solution NMR

**Figure 4:**
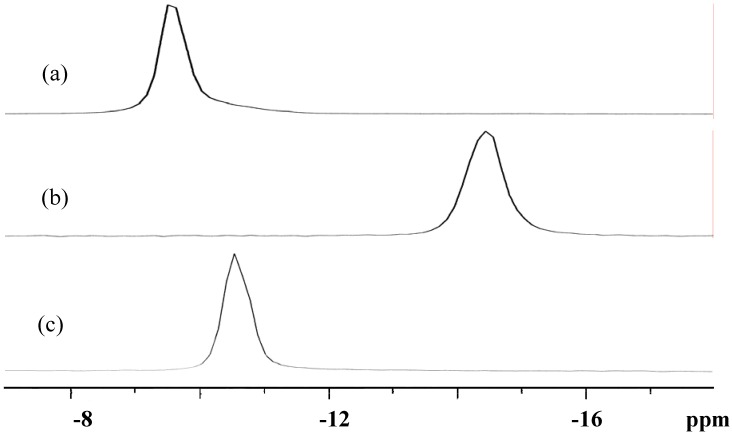
Solid-state ^31^P-CPMAS NMR spectra of (a) K_5_H[[{Ti(H_2_O)_3_}_2_(μ-O)](α-PW_9_Ti_2_O_38_)]_2_·9H_2_O (**K-1**), (b) K_3_[{Ti_4_(μ-O)_3_(SO_4_)_2_(H_2_O)_8_}(α-PW_9_O_34_)]·6H_2_O (**K-2**) and (c) K_10_H_2_[(α-1,2,3-PW_9_Ti_3_O_37_)_2_O_3_]·15H_2_O as a precursor.

**Figure 5:**
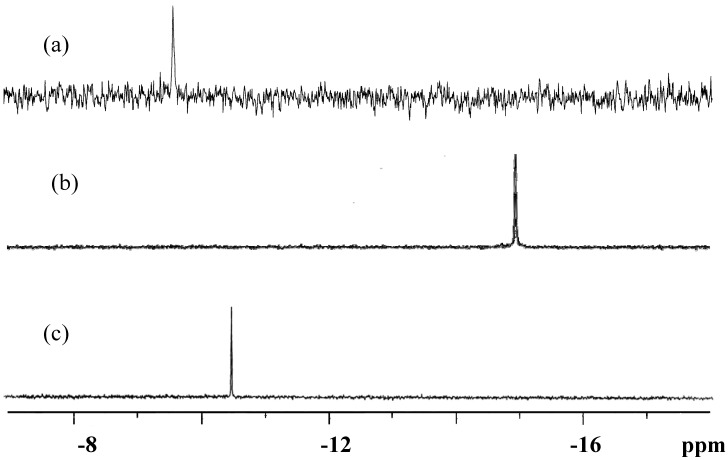
^31^P-NMR spectra of (a) K_5_H[[{Ti(H_2_O)_3_}_2_(μ-O)](α-PW_9_Ti_2_O_38_)]_2_·9H_2_O (**K-1**) in 0.1 M aqueous HCl, (b) K_3_[{Ti_4_(μ-O)_3_(SO_4_)_2_(H_2_O)_8_}(α-PW_9_O_34_)]·6H_2_O (**K-2**) in aqueous 0.5 M aqueous H_2_SO_4_, and (c) K_10_H_2_[(α-1,2,3-PW_9_Ti_3_O_37_)_2_O_3_]·15H_2_O as a precursor in D_2_O.

Solid-state ^31^P-CPMAS NMR of **K-1**, **K-2** and the precursor (Figure 4) showed a single resonance at −9.54, −14.43 and −10.64 ppm, respectively; **K-1** and the precursor should correspond to the dimeric structures determined by X-ray analysis, and **K-2** to the monomeric structure. On the other hand, solution ^31^P-NMR spectra of **K-1**, **K-2** and the precursor in D_2_O (Figure 5) showed only one resonance at −9.70, −14.96 and −10.52 ppm, respectively, confirming the formation of a single phosphorus-containing compound. The dimeric nature in aqueous solution of the precursor has been confirmed with the molecular weight measurements based on ultracentrifugation sedimentation equilibrium [12]. ^31^P-NMR of **K-1** in 0.1 M HCl aqueous solution observed at −9.61 ppm, and those of **K-2** in 0.5 M H_2_SO_4_ and 1 M HNO_3_ aqueous solutions observed at −15.07 and −14.86 ppm, respectively, were almost the same as the ^31^P-NMR spectra observed in D_2_O.

Solid-state and solution ^31^P-NMR suggest that the dimeric structure of **1** and the monomeric structure of **2** will be maintained in aqueous solution. However, it is not clear whether the coordinated sulfate ions in the solid state are kept in aqueous solution.

^183^W-NMR measurement in HCl-acidic aqueous solution of **K-1** was unsuccessful, because not enough concentration was obtained for the measurement. In order to increase the solubility of **1**, the cation exchange was attempted with a batch method using the cation-exchange resin column in Na^+^ form and, also, with direct cation exchange using excess amount of NaClO_4_. However, compound **K-1** seriously decomposed. On the other hand, ^183^W-NMR of **K-2** in 0.5 M aqueous H_2_SO_4_ solution was successfully measured (Figure S1). In comparison with the ^183^W-NMR of the precursor, it is clear that the monomeric species is present in aqueous solution, also as suggested by solution ^31^P-NMR, although it is unclear if the solid-state structure containing two coordinated sulfate ions is kept.

## 4. Conclusions

The dimeric species linked through three intermolecular Ti-O-Ti bonds of the two tri-titanium(IV)-substituted α-Keggin polyoxometalate (POM) subunits, K_10_H_2_[(α-1,2,3-PW_9_Ti_3_O_37_)_2_O_3_]·15H_2_O, has been considered as a very stable form of the Ti-substituted Keggin POMs, because the monomeric form has been difficult to derive [12]. Nevertheless, this POM has shown effective epoxidation catalysis of olefins with aqueous hydrogen peroxide [19]. In this work, a new reaction of this POM with Ti(SO_4_)_2_·4H_2_O was found. Depending upon reaction conditions, it gave the two new Ti-containing products; one was obtained as K_5_H[[{Ti(H_2_O)_3_}_2_(μ-O)](α-PW_9_Ti_2_O_38_)]_2_·9H_2_O (**K-1**) by the 10 min-reaction at 60 °C, while the other was obtained as K_3_[{Ti_4_(μ-O)_3_(SO_4_)_2_(H_2_O)_8_}(α-PW_9_O_34_)]·6H_2_O (**K-2**) by the 30 min-reaction at 80°C. Interestingly, the former product contained a dimeric species of the tetra-titanium(IV) substituted Keggin units via two intermolecular Ti-O-Ti bonds, while the latter contained a monomeric tetra-titanium(IV) oxide cluster constructed on the tri-lacunary Keggin unit. In the solid state, the latter contained two coordinated sulfate ions. These compounds have been unequivocally characterized in the solid state and in solution. In the viewpoint of host-guest chemistry of Ti-substitution in POM, the Ti_4_ center in **1** was composed of a combination of one host-one guest and one host-two guest coordination, whereas the Ti_4_ center in **2** comprised a new type of host-guest relation, *i.e.*, three host-four guest coordination. The polyoxoanions **1** and **2** are also of interest as possible solid oxidation catalysts. Studies in this direction are in progress.

## Supplementary Materials

Supplementary File 1

Supplementary File 2

Supplementary material (solution ^183^W-NMR of **2** and the precursor (Figure S1); bond lengths (Å) and angles (°) for **1** and **2** (Tables S1 and S2); bond valence sum (BVS) calculations for **1** and **2** (Table S3 and S4)) can be downloaded online at http://www.mdpi.com/1996-1944/3/1/503/s1. CSD reference numbers 421237 for **K-1** and 421238 for **K-2**. For crystallographic data in CIF or other electronic format, see http://www.mdpi.com/1996-1944/3/1/503/s2.
