# Supplementary material for: Transformation of Tri-Titanium(IV)-Substituted α-Keggin Polyoxometalate (POM) into Tetra-Titanium(IV)-Substituted POMs : Reaction Products of Titanium(IV) Sulfate with the Dimeric Keggin POM Precursor under Acidic Conditions
Source: Materials (Basel). 2010 Jan 15;3(1):503–18. doi: 10.3390/ma3010503 (PMC5525177; doi:10.3390/ma3010503)
Supplement: Supplementary File 1 [file materials-03-00503-s001.pdf]

# Transformation of Tri-Titanium(IV)-Substituted α-Keggin Polyoxometalate (POM) into Tetra-Titanium(IV)-Substituted POMs: Reaction Products of Titanium(IV) Sulfate with the Dimeric Keggin POM Precursor under Acidic Conditions

**Yuki Mouri, Yoshitaka Sakai, Yoshitaka Kobayashi, Shoko Yoshida and Kenji Nomiya ***

**Figure S1.** $^{183}$W-NMR spectra of (a) $K_3[\{Ti_4(\mu\text{-}O)_3(SO_4)_2(H_2O)_8\}(\alpha\text{-}PW_9O_{34})]\cdot 6H_2O$ (**K-2**) in 0.5 M aqueous $H_2SO_4$, and (b) $K_{10}H_2[(\alpha\text{-}1,2,3\text{-}PW_9Ti_3O_{37})_2O_3]\cdot 15H_2O$ as a precursor in $D_2O$.

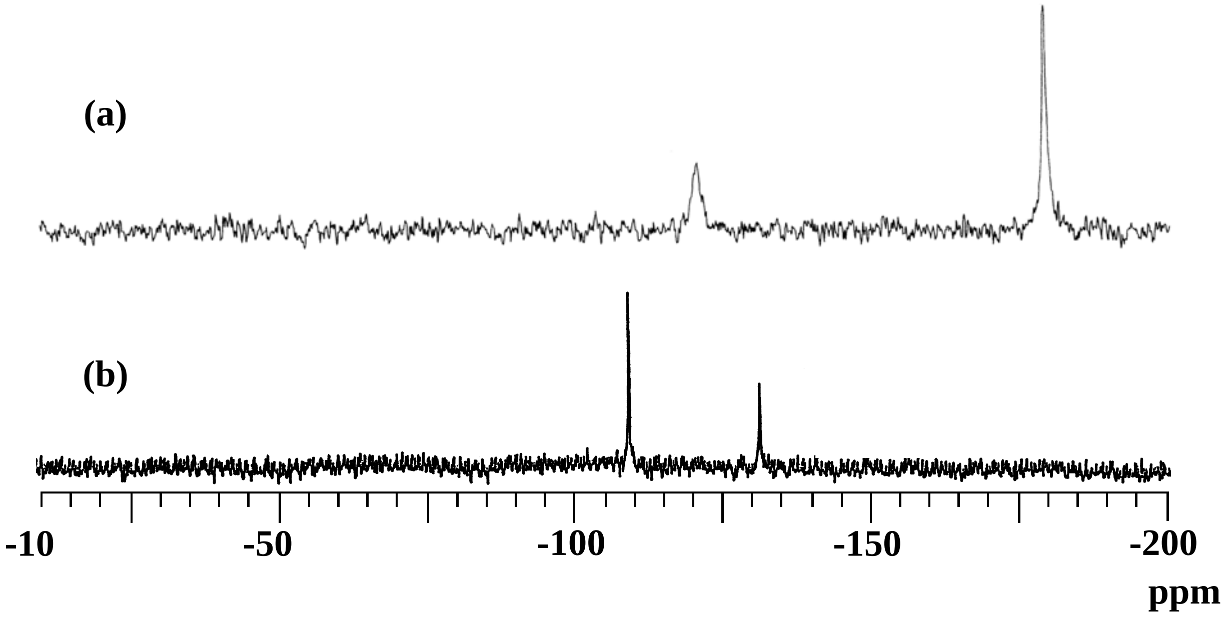


**Table S1.** Bond lengths (Å) and angles ($^{o}$) for **1**.

| | | |
|---|---|---|
| **W-O$_t$ (O$_t$: terminal oxygen)** | | |
| W(1)-O(1) | 1.710(10) | |
| W(2)-O(2) | 1.715(11) | |
| W(3)-O(3) | 1.719(11) | |
| W(4)-O(13) | 1.717(9) | |
| W(5)-O(14) | 1.709(10) | |
| W(6)-O(15) | 1.713(11) | |
| W(7)-O(16) | 1.719(9) | |
| W(8)-O(17) | 1.706(11) | |
| W(9)-O(18) | 1.708(10) | |
| | | average = 1.713 [1.706(11)-1.719(10)] |

**Table S1.** *Cont.*

| | | |
|---|---|---|
| **W-$O_c$ ($O_c$: corner-sharing oxygen)** | | |
| W(1)-O(7) | 1.856(9) | |
| W(1)-O(12) | 1.903(10) | |
| W(2)-O(8) | 1.889(10) | |
| W(2)-O(9) | 1.861(10) | |
| W(3)-O(10) | 1.910(10) | |
| W(3)-O(11) | 1.862(11) | |
| W(4)-O(24) | 1.894(9) | |
| W(4)-O(7) | 1.965(9) | |
| W(5)-O(20) | 1.915(9) | |
| W(5)-O(8) | 1.972(9) | |
| W(6)-O(20) | 1.905(9) | |
| W(6)-O(9) | 1.976(10) | |
| W(7)-O(22) | 1.930(10) | |
| W(7)-O(10) | 1.942(10) | |
| W(8)-O(22) | 1.877(10) | |
| W(8)-O(11) | 1.984(10) | |
| W(9)-O(24) | 1.923(9) | |
| W(9)-O(12) | 1.927(9) | |
| | | average = 1.916 [1.856(9)-1.984(10)] |
| **W-Oc (Oc: corner-sharing oxygen coordinating to Ti atom)** | | |
| W(7)-O(28) | 1.848(9) | |
| W(6)-O(27) | 1.832(9) | |
| | | average = 1.840 [1.832(9)-1.848(9)] |
| **W-$O_e$ ($O_e$: edge-sharing oxygen)** | | |
| W(1)-O(4) | 1.902(10) | |
| W(1)-O(6) | 1.941(10) | |
| W(2)-O(5) | 1.909(11) | |
| W(2)-O(4) | 1.949(10) | |
| W(3)-O(6) | 1.915(10) | |
| W(3)-O(5) | 1.955(11) | |
| W(4)-O(19) | 1.944(9) | |
| W(5)-O(19) | 1.926(9) | |
| W(6)-O(21) | 1.926(10) | |
| W(7)-O(21) | 1.914(11) | |
| W(8)-O(23) | 1.952(10) | |
| W(9)-O(23) | 1.897(9) | |
| | | average = 1.928 [1.897(9)-1.955(11)] |

**Table S1.** *Cont.*

| | | |
|---|---|---|
| **W-O$_e$ (O$_e$: edge-sharing oxygen coordinating to Ti atom)** | | |
| W(4)-O(25) | 1.839(8) | |
| W(5)-O(26) | 1.828(9) | |
| W(8)-O(29) | 1.817(9) | |
| W(9)-O(30) | 1.866(9) | |
| | | average = 1.838 [1.817(9)-1.866(9)] |
| **W-O$_a$ (oxygen coordinating to P atom)** | | |
| W(1)-O(37) | 2.423(9) | |
| W(2)-O(37) | 2.423(10) | |
| W(3)-O(37) | 2.411(9) | |
| W(4)-O(34) | 2.498(9) | |
| W(5)-O(34) | 2.380(9) | |
| W(6)-O(35) | 2.377(10) | |
| W(7)-O(35) | 2.355(9) | |
| W(8)-O(36) | 2.455(8) | |
| W(9)-O(36) | 2.489(9) | |
| | | average = 2.423 [2.355(9)-2.498(9)] |
| **P-O** | | |
| P(1)-O(34) | 1.545(9) | |
| P(1)-O(35) | 1.518(9) | |
| P(1)-O(36) | 1.544(9) | |
| P(1)-O(37) | 1.557(9) | |
| | | average = 1.541 [1.518(9)-1.557(9)] |
| **O-P-O** | | |
| O(34)-P(1)-O(35) | 110.7(5) | |
| O(34)-P(1)-O(36) | 109.7(5) | |
| O(34)-P(1)-O(37) | 108.4(5) | |
| O(35)-P(1)-O(36) | 110.4(5) | |
| O(35)-P(1)-O(37) | 109.4(5) | |
| O(36)-P(1)-O(37) | 108.1(5) | |
| | | average = 109.5 [108.1(5)-110.7(5)] |

**Table S2.** Bond lengths (Å) and angles ($^o$) for **2**

| | |
|---|---|
| **W-O$_t$ (O$_t$: terminal oxygen)** | |
| W(1)-O(1) | 1.716(6) |
| W(2)-O(2) | 1.717(6) |
| W(3)-O(3) | 1.706(6) |
| W(4)-O(13) | 1.708(6) |
| W(5)-O(14) | 1.719(6) |
| W(6)-O(15) | 1.719(6) |
| W(7)-O(16) | 1.729(5) |
| W(8)-O(17) | 1.713(6) |
| W(9)-O(18) | 1.719(6) |
| | average = 1.716 [1.706(6)-1.729(5)] |
| **W-O$_c$ (O$_c$: corner-sharing oxygen)** | |
| W(1)-O(7) | 1.877(6) |
| W(1)-O(12) | 1.875(6) |
| W(2)-O(8) | 1.932(6) |
| W(2)-O(9) | 1.856(6) |
| W(3)-O(10) | 1.884(5) |
| W(3)-O(11) | 1.845(6) |
| W(4)-O(24) | 1.879(6) |
| W(4)-O(7) | 1.960(6) |
| W(5)-O(20) | 1.943(5) |
| W(5)-O(8) | 1.884(6) |
| W(6)-O(20) | 1.872(5) |
| W(6)-O(9) | 1.995(6) |
| W(7)-O(22) | 1.947(5) |
| W(7)-O(10) | 1.933(5) |
| W(8)-O(22) | 1.877(5) |
| W(8)-O(11) | 1.984(6) |
| W(9)-O(24) | 1.920(6) |
| W(9)-O(12) | 1.966(6) |
| | average = 1.913 [1.845(6)-1.995(6)] |
| **W-O$_c$ (O$_c$: corner-sharing oxygen coordinating to Ti atoms)** | |
| W(4)-O(25) | 1.862(6) |
| W(5)-O(26) | 1.932(6) |
| W(6)-O(27) | 1.843(5) |
| W(7)-O(28) | 1.856(5) |
| W(8)-O(29) | 1.855(6) |
| W(9)-O(30) | 1.862(6) |
| | average = 1.868 [1.843(5)-1.932(6)] |

**Table S2.** *Cont.*

| | | | |
|---|---|---|---|
| **W-O$_e$ (O$_e$: edge-sharing oxygen)** | | | |
| W(1)-O(4) | 1.930(6) | W(8)-O(23) | 1.926(6) |
| W(1)-O(6) | 1.942(6) | W(9)-O(23) | 1.908(6) |
| W(2)-O(5) | 1.907(6) | | |
| W(2)-O(4) | 1.961(6) | | |
| W(3)-O(6) | 1.943(6) | | |
| W(3)-O(5) | 1.966(6) | | |
| W(4)-O(19) | 1.934(6) | | |
| W(5)-O(19) | 1.903(6) | | |
| W(6)-O(21) | 1.946(5) | | |
| W(7)-O(21) | 1.884(5) | | |
| | | average = 1.929 [1.884(5)-1.966(6)] | |
| **W-O$_a$ (oxygen coordinating to P atom)** | | | |
| W(1)-O(34) | 2.354(5) | | |
| W(2)-O(34) | 2.380(5) | | |
| W(3)-O(34) | 2.364(5) | | |
| W(4)-O(31) | 2.394(5) | | |
| W(5)-O(31) | 2.308(5) | | |
| W(6)-O(32) | 2.378(5) | | |
| W(7)-O(32) | 2.332(5) | | |
| W(8)-O(33) | 2.377(5) | | |
| W(9)-O(33) | 2.340(5) | | |
| | | average = 2.359 [2.308(5)-2.394(5)] | |
| **P-O** | | | |
| P(1)-O(31) | 1.535(5) | | |
| P(1)-O(32) | 1.521(5) | | |
| P(1)-O(33) | 1.528(5) | | |
| P(1)-O(34) | 1.582(5) | | |
| | | average = 1.542 [1.521(5)-1.582(5)] | |
| **S-O** | | | |
| S(1)-O(1X) | 1.489(6) | S(2)-O(5X) | 1.505(5) |
| S(1)-O(2X) | 1.470(6) | S(2)-O(6X) | 1.489(6) |
| S(1)-O(3X) | 1.500(6) | S(2)-O(7X) | 1.474(6) |
| S(1)-O(4X) | 1.455(6) | S(2)-O(8X) | 1.456(6) |
| | | average = 1.480 [1.455(6)-1.505(5)] | |
| **O-P-O** | | | |
| O(31)-P(1)-O(32) | 112.2(3) | | |
| O(31)-P(1)-O(33) | 112.2(3) | | |
| O(31)-P(1)-O(34) | 106.9(3) | | |
| O(32)-P(1)-O(33) | 111.9(3) | | |
| O(32)-P(1)-O(34) | 106.6(3) | | |
| O(33)-P(1)-O(34) | 106.7(3) | | |
| | | average = 109.4 [106.6(3)-112.2(3)] | |

**Table S2.** *Cont.*

| | | | |
|---|---|---|---|
| **O-S-O** | | | |
| O(1X)-S(1)-O(2X) | 109.3(4) | O(4X)-S(1)-O(5X) | 110.2(3) |
| O(1X)-S(1)-O(3X) | 109.7(3) | O(4X)-S(1)-O(6X) | 107.7(3) |
| O(1X)-S(1)-O(7X) | 108.7(4) | O(4X)-S(1)-O(8X) | 107.9(3) |
| O(2X)-S(1)-O(3X) | 109.5(4) | O(5X)-S(1)-O(6X) | 109.5(3) |
| O(2X)-S(1)-O(7X) | 111.0(4) | O(5X)-S(1)-O(8X) | 110.4(3) |
| O(3X)-S(1)-O(7X) | 108.7(4) | O(6X)-S(1)-O(8X) | 111.1(4) |
| | | average = 109.5 [107.7(3)-111.1(4)] | |

**Table S3.** Bond valence sum (BVS) calculations of W, Ti, O and P atoms for **K-1.**

| | | | | | |
|---|---|---|---|---|---|
| O(1) | 1.750 | O(32) | 1.951 | W(1) | 6.201 |
| O(2) | 1.726 | O(33) | 1.965 | W(2) | 6.162 |
| O(3) | 1.708 | O(34) | 1.925 | W(3) | 6.057 |
| O(4) | 1.958 | O(35) | 1.901 | W(4) | 6.032 |
| O(5) | 1.924 | O(36) | 1.939 | W(5) | 6.155 |
| O(6) | 1.942 | O(37) | 1.949 | W(6) | 6.144 |
| O(7) | 2.057 | O(38) | 1.980 | W(7) | 6.127 |
| O(8) | 1.941 | | | W(8) | 6.171 |
| O(9) | 2.016 | O(1W) | 0.489 | W(9) | 6.133 |
| O(10) | 1.954 | O(2W) | 0.374 | | |
| O(11) | 1.994 | O(3W) | 0.462 | Ti(1) | 4.178 |
| O(12) | 2.012 | O(4W) | 0.440 | Ti(2) | 4.202 |
| O(13) | 1.717 | O(5W) | 0.391 | Ti(3) | 4.088 |
| O(14) | 1.754 | O(6W) | 0.467 | Ti(4) | 4.236 |
| O(15) | 1.736 | | | | |
| O(16) | 1.708 | | | P(1) | 4.916 |
| O(17) | 1.769 | | | | |
| O(18) | 1.759 | | | | |
| O(19) | 1.906 | | | | |
| O(20) | 2.038 | | | | |
| O(21) | 1.984 | | | | |
| O(22) | 2.079 | | | | |
| O(23) | 1.966 | | | | |
| O(24) | 2.048 | | | | |
| O(25) | 1.819 | | | | |
| O(26) | 1.877 | | | | |
| O(27) | 2.038 | | | | |
| O(28) | 2.065 | | | | |
| O(29) | 1.836 | | | | |
| O(30) | 1.799 | | | | |
| O(31) | 2.012 | | | | |

**Table S4.** Bond valence sum (BVS) calculations of W, Ti, S, O and P atoms for **K-2.**

| | | | | | |
|---|---|---|---|---|---|
| O(1) | 1.722 | O(32) | 1.910 | W(1) | 6.163 |
| O(2) | 1.717 | O(33) | 1.879 | W(2) | 6.057 |
| O(3) | 1.769 | O(34) | 1.991 | W(3) | 6.184 |
| O(4) | 1.853 | | | W(4) | 6.147 |
| O(5) | 1.903 | O(1M) | 2.046 | W(5) | 6.080 |
| O(6) | 1.867 | O(2M) | 2.089 | W(6) | 6.111 |
| O(7) | 2.004 | O(3M) | 2.080 | W(7) | 6.140 |
| O(8) | 2.053 | | | W(8) | 6.130 |
| O(9) | 1.989 | O(1W) | 0.400 | W(9) | 6.080 |
| O(10) | 2.051 | O(2W) | 0.463 | | |
| O(11) | 2.049 | O(3W) | 0.467 | Ti(1) | 4.210 |
| O(12) | 1.996 | O(4W) | 0.417 | Ti(2) | 4.238 |
| O(13) | 1.759 | O(5W) | 0.421 | Ti(3) | 4.177 |
| O(14) | 1.708 | O(6W) | 0.434 | Ti(4) | 4.257 |
| O(15) | 1.708 | O(7W) | 0.387 | | |
| O(16) | 1.662 | O(8W) | 0.478 | P(1) | 4.915 |
| O(17) | 1.736 | | | | |
| O(18) | 1.708 | O(1X) | 1.943 | S(1) | 5.933 |
| O(19) | 1.994 | O(2X) | 1.516 | S(2) | 5.894 |
| O(20) | 2.061 | O(3X) | 1.884 | | |
| O(21) | 2.018 | O(4X) | 1.905 | | |
| O(22) | 2.036 | O(5X) | 1.900 | | |
| O(23) | 2.001 | O(6X) | 1.500 | | |
| O(24) | 2.100 | O(7X) | 1.579 | | |
| O(25) | 2.020 | O(8X) | 1.575 | | |
| O(26) | 1.965 | | | | |
| O(27) | 2.050 | | | | |
| O(28) | 2.000 | | | | |
| O(29) | 2.037 | | | | |
| O(30) | 2.045 | | | | |
| O(31) | 1.871 | | | | |
